# Differences in Covid-19 mortality among persons 70 years and older in an integrated care setting in region Stockholm: a multi-level analysis between March 2020-February 2021

**DOI:** 10.1186/s12889-024-17904-4

**Published:** 2024-02-14

**Authors:** Megan Doheny, Antonio Ponce de Leon, Bo Burström, Ann Liljas, Janne Agerholm

**Affiliations:** 1 Aging Research Center, Karolinska Institutet, Stockholm, Sweden; 2 Department of Global Public Health, Karolinska Institutet, Stockholm, Sweden; 3 Centre for Epidemiology and Community Medicine, Stockholm County Council, Stockholm, Sweden; 4 Department of Neurobiology, Care Sciences and Society, Karolinska Institutet, Aging Research Center, Tomtebodavägen 18A, plan 9, Stockholm, 171 65 Sweden

**Keywords:** Integrated care, Covid-19 mortality, Municipal social care, Care organisation, Multi-level model

## Abstract

**Background:**

In Norrtälje municipality, within Region Stockholm, there is a joint integrated care organisation providing health and social care, which may have facilitated a more coordinated response to the covid-19 pandemic compared to the otherwise decentralised Swedish system. This study compares the risk of covid-19 mortality among persons 70 years and older, in the municipalities of Stockholm, Södertälje, and Norrtälje, while considering area and individual risk factors.

**Methods:**

A population-based study using linked register data to examine covid-19 mortality among those 70 + years (*N* = 127,575) within the municipalities of interest between the periods March-August 2020 and September 2020-February 2021. The effect of individual and area level variables on covid-19 mortality among inhabitants in 68 catchment areas were examined using multi-level logistic models.

**Results:**

Individual factors associated with covid-19 mortality were sex, older age, primary education, country of birth and poorer health as indicated by the Charlson Co-morbidity Index. The area-level variables associated were high deprivation (OR: 1.56, CI: 1.18–2.08), population density (OR: 1.14, CI: 1.08–1.21), and usual care. Together, this explained 85.7% of the variation between catchment areas in period 1 and most variation was due to individual risk factors in period 2. Little of the residual variation was attributed to differences between catchment areas.

**Conclusion:**

Integrated care in Norrtälje may have facilitated a more coordinated response during period 1, compared to municipalities with usual care. In the future, integrated care should be considered as an approach to better protect and meet the care needs of older people during emergency situations.

**Supplementary Information:**

The online version contains supplementary material available at 10.1186/s12889-024-17904-4.

## Introduction

During the coronavirus disease (covid-19) pandemic all age groups were at risk of contracting the virus, however, older age was associated with an increased risk of severe disease, hospitalisation, and mortality [1]. Moreover, variations were observed among older ages that were dependent on health problems and social care needs [2]. The highest mortality rates were observed among older persons receiving home-help services or living in care homes during the first wave of the covid-19 pandemic [3, 4]. In Sweden, 28.4% of deaths in covid-19 were among persons receiving home-help services in their own homes and 50.6% among care homes residents [2]. In Region Stockholm covid-19 mortality varied between municipalities and geographic areas and by time [5].

Other individual factors associated with an increased risk of covid-19 mortality were male sex, country of birth, and lower socio-economic position (SEP) indicated by education and co-morbidities [6–8]. Moreover, previous studies identified contextual factors that increased the community spread of the virus, including high population density, urban areas, and socio-economically disadvantaged area [9]. Further, these contextual factors have been associated with an increased risk of covid-19 related hospitalisations and mortality among those 18 years and older in a previous study set in Stockholm [10]. Similarly, a Swedish ecological geospatial study observed contextual factors such as high population density and a greater proportion of migrants, increased the risk of covid-19 related hospitalizations and mortality. In another study, contrarily, areas with a high proportion of persons 65 years and older had a reduced risk of covid-19 related hospitalisations and mortality [9, 10].

In Sweden, there are universal tax-funded health and social care systems, however, the provision is decentralised and fragmented, as the financing and organization of care is separated. The 21 regions are responsible for the provision of medical and health care services, while the 290 municipalities are responsible for providing social care to older people, including both home-help services (domestic and personal care) and the management of care homes. The administrative division between care systems limits the continuity and coordination of care to persons in need under normal circumstances. However, when this division of responsibility is extended to crisis management, such as the covid-19 pandemic, this has had implications for patient safety and quality of care [11, 12]. This division caused problems in the care of older people with covid-19, which was highlighted in a report by the national Corona Commission [13].

During the first wave, measures were introduced to limit the community spread of the virus, and to protect the healthcare system and maintain bed capacity for the inflow of severely ill covid-19 patients. Healthcare workers were prioritized in the allocation of personal protective equipment (PPE) and trained in measures meant to limit the spread of infectious disease. Therefore, at the initial stages the social care system for older persons were not prioritized and PPE was not widely distributed to those working in home-help services or in care homes [2, 4]. There was poor coordination between systems in response to the first wave of the covid-19 pandemic, combined with a limited ability to share information between health and social care systems, which placed vulnerable groups at greater risk of infection and severe outcomes [2].

The municipality Norrtälje, located in the north of Stockholm County at some distance from more central areas, implemented an integrated care (IC) approach in 2006, in contrast to the fragmented organisation of health and social care in the rest of region Stockholm. The IC approach began with pooled health and social care budgets and the formation of a joint board for health and social care which included representatives from the municipality and region. The board purchases services from a jointly owned company, Vårdbolaget Tiohundra which is the main provider of both health and social care in Norrtälje. The implementation and development of IC has been described in detail elsewhere [14, 15]. The IC approach led to organisational changes being introduced to improve the lines of communication and information sharing between health and social care providers [15, 16]. These organisational changes may have been beneficial in coordinating the response to the covid-19 pandemic by health and social care personnel, in terms of sharing of information, resources and coordination of care for risk groups such as those with multiple health problems and social care needs.

This study aimed to compare the risk of covid-19 mortality among persons 70 years and older, in different municipalities Stockholm, Södertälje, and Norrtälje, considering contextual and individual risk factors. The municipalities of Stockholm and Södertälje were selected as comparison, to consider the different municipal responses to the covid-19 pandemic, as both municipalities are examples of the usual decentralised health-and-social care organisation. Stockholm was chosen because it is the largest, centrally located and socio-economically heterogenous municipality. Södertälje was selected because of its similarities to Norrtälje, with a similar population size and socio-economic composition as Norrtälje, except with a larger proportion of inhabitants born outside Sweden.

## Methods

A population-based study using register data linked via encrypted serial numbers. The study population was derived from the Total Population Register *“Registret över Totalbefolkning*’ (RTB), a register which contains information on life events such as births, deaths, and migration [17]. The RTB included all persons 18 years and older living in Stockholm County between 2019 and 2020 *N* = 1,855,404), of which there was *N* = 277,417 persons 70 years and older on the 31st of December 2019. The final study population included only those 70 years and older registered as living in the municipalities of Norrtälje, Södertälje and Stockholm (*N* = 127,575). The study was divided into two periods: period 1 between March 1st and August 31st, 2020, and period 2 between September 1st 2020 and February 28th 2021. Period 1 indicates the first wave and initial response to the covid-19 by the health and social care system, while period 2 is defined by the second wave prior to widespread availability of vaccinations [18]. In period 2, there were (*N* = 123,622) excluding (*n* = 3,953) persons who died during period 1.

### Outcomes

The Swedish Cause of Death Register was used to distinguish between covid-19 related from other causes of mortality. At the beginning, covid-19-mortality was officially defined as deaths occurring in patients who tested positive for SARS-CoV-2 through an RT-PCR test. However, this definition was later expanded to include covid-19 related deaths without a positive test. The International Classification of Disease (ICD-10) codes used to indicate covid-19 related mortality (U07.1, U07.2, U07.3) based on recommendations by the National Board of Health and Welfare (NBHW) [19].

### Individual variables

Individual variables were obtained from RTB; sex was categorised into male or female; age measured using year of birth and categorized into five-year intervals. Country of birth was categorised as born in Sweden or outside of Sweden. The data was linked to the Longitudinal Integrated Database for Social Insurance and Labour Market Studies (LISA), where measures of SEP were obtained. The LISA register is a collection of variables from several different population-based registers which are individually linked [20]. Income was measured using net annual equalised household income in 2019 and was ranked into quintiles with group 1 (lowest) and group 5 (highest), based on the distribution of income in the study population. Education level was categorised according to the Swedish educational system into years of education: primary (< 9 years), secondary (9–12 years) and post-secondary (> 12 years).

Municipal social care use was measured in the Swedish Social Services Register which collects monthly data on individual use of home-help (domestic and/or personal care) services and care home residence [21]. Municipal social care use was categorized into living in own homes without home-help, living in own home with home-help, and care-home resident, during period 1 and/or period 2.

The Charlson Comorbidity Index (CCI) was included as an indicator of health status. The CCI score was measured in the National Patient Register (NPR) using inpatient and outpatient care diagnoses between 2016 and 2018. The CCI assigns scores ranging from 1 to 6 to different morbidities according to the severity of disease. The weighted score was calculated for everyone in the study population and was used to adjust for morbidity in the analysis [22].

### Area-level variables

The area-level was delineated by catchment area *(“betjäningsområde”)* within each municipality, previously used to denote service areas which primary care centres were responsible for. The catchment areas include 7 areas in Norrtälje, 52 areas in Stockholm municipality and 9 areas in Södertälje (see table S1). A variable indicating organisation of care in the municipality was included (IC), where Norrtälje was the reference category which was compared to usual care in Stockholm and Södertälje. Additionally, area variables were calculated based on all inhabitants 18 years and older, including a composite neighbourhood deprivation score (NDS) which was divided into three levels from the least to the most deprived, the NDS was generated to adjust for the socio-economic circumstances of areas in Stockholm County and is described in detail by Bell et al. [10]. Population density was calculated based on the number of inhabitants per square kilometre and was included in the model as a continuous variable.

### Analysis

The data had a multi-level structure with level one consisting of individuals residing in the municipalities of interest and were nested within the catchment areas within each municipality [23]. Multivariate logistic regression models used to select the individual-level covariates and goodness of fit was assessed based on plots of the predicted observed values and the pseudo r-squared (see figure S1 and S2).

The multi-level logistic models were specified as follows: model 0 included the random intercept term and provides information on the individual variance in how the probability of covid-19 mortality was distributed across the different catchment areas. Model 1 included the individual covariates selected from the multivariate models, to adjust for the individual composition of the catchment areas. Model 2 included model 1, and a selection of area-level variables (see table S3-S5), this provided information about the association between area-level variables and covid-19 mortality among individuals within the catchment areas. Model 3 expanded upon model 2, to evaluate the specific contextual effects of IC, and the organization of care in the municipality was included as an area-level variable.

Finally, in model 4 the use of municipal social care was included in the fixed effects, considering the potential mediation pathway between the organisation of care (integrated vs. usual) and municipal social care [24]. The variance and the proportional change in the variance (PCV) were estimated to compare each model. Caterpillar plots were used to visually assess and compare the variance between catchment areas across the distribution in period 1 and 2.

The amount of explanatory power attributed to contextual effects was estimated using the variance partition coefficient (VPC), that represents the proportion of variability in covid-19 mortality that is attributable to systematic differences between areas [25]. The VPC was estimated using a simulation method based on a probability scale of a range explanatory variables, from the models described (see table S6) [26].

The regression coefficients were estimated using generalised mixed effects models with a maximum likelihood estimation and a binomial distribution expressed as odds ratios and confidence intervals (R package Lme4).

## Results

Among the *N* = 127,575 inhabitants 70 years and older in the municipalities of Norrtälje, Södertälje and Stockholm there was a total of 1,674 covid-19 related deaths between 1st March 2020 and 28th February 2021, with more covid-19 deaths in period 1 compared to period 2. Age standardized rates of covid-19 mortality stratified by municipality and care groups are shown in Table 1. The covid-19 mortality rate was higher in Södertälje, and Stockholm municipality compared to Norrtälje (3.1 covid-19 deaths per 10,000 persons) in period 1. Home-help users and care home residents had the highest rate of covid-19 mortality in both periods. In period 2, the rate of covid-19 mortality increased in Norrtälje and decreased in the other municipalities compared to period 1.

**Table 1:** Description of age standardized covid-19 mortality during period 1 and 2 within the municipalities of interest and municipal social care

**Period 1**	**Norrtälje**	**Södertälje**	**Stockholm**
	*N* = 12,657	*N* = 11,637	*N* = 103,281
No. Deaths	305	382	3,266
(%) of covid-19 deaths	8.5%	29.0%	28.3%
**covid-19 mortality rate per 10,000 among….**			
All inhabitants	3.1	13.6	12.4
Inhabitants independent in the community	1.1	3.8	3.7
Inhabitants with home-help in the community	6.4	34.0	20.7
Inhabitants living in care homes	29.1	79.9	84.7
**Period 2**	**Norrtälje**	**Södertälje**	**Stockholm**
	*N* = 12,352	*N* = 11,255	N-100,015
No. Deaths	381	357	2,598
(%) of covid-19 deaths	16.8%	22.7%	18.0%
**covid-19 mortality rate per 10,000 among…**			
All inhabitants	7.9	10.7	6.7
Inhabitants independent in the community	3.5	3.0	2.6
Inhabitants with home-help in the community	14.5	28.2	9.5
Inhabitants living in care homes	30.9	54.4	36.6

*Age standardized mortality rates calculated based on European standard population weights

*No. of deaths = number of deaths due to all-causes

*(%) proportion of deaths with covid-19 as underlying cause of death

Table 2 describes the composition of the study population. The age distribution did not vary between the municipalities. In Norrtälje, 47.9% of inhabitants were male compared to 43.7% in Södertälje and 42.5% in Stockholm municipality. Södertälje had the highest proportion of inhabitants born outside of Sweden (34%), compared to 10.5% in Norrtälje. There was a higher proportion of inhabitants in lower income groups and with primary education in Norrtälje and Södertälje, and there were slight differences in morbidity based on the CCI score and risk group diagnosis between the municipalities. In period 1, in Södertälje 14.5% of inhabitants were receiving home-help services compared to 13.8% in Stockholm and 11.3% in Norrtälje. The proportion of care home residents was similar (6.3% and 6.2%) in Norrtälje and Stockholm and 5.7% in Södertälje.

**Table 2:** Socio-demographic composition of the inhabitants 70 years and older in Norrtälje, Södertälje and Stockholm municipality in period 1

	Integrated Care	Usual Care	
	Norrtälje	Södertälje	Stockholm
	*N* = 12,657	*N* = 11,637	*N* = 103,281
**Age group**			
70–74 years	37.9% (4,799)	35.4% (4,120)	37.9% (39,187)
75–79 years	29.5% (3,7f34)	29.2% (3,393)	27.6% (28,489)
80–84 years	17.0% (2,152)	19.0% (2,211)	16.2% (16,736)
85–89 years	9.7% (1,231)	10.5% (1,228)	10.3% (10,598)
90–94 years	4.6% (587)	4.5% (522)	5.8% (5,975)
95 years and older	1.2% (154)	1.4% (163)	2.2% (2,314)
**Sex** (male)	47.9% (6,056)	43.7% (5,080)	42.5% (43,852)
**Country of birth** (outside of Sweden)	10.5% (1,333)	34.0% (3,951)	20.7% (21,395)
**Level of Education**			
Primary	32.1% (4,061)	32.8% (3,818)	19.6% (20,279)
Secondary	46.2% (5,846)	40.0% (4,659)	39.2% (40,494)
Tertiary	20.7% (2,626)	19.7% (2,298)	38.6% (39,884)
Missing	1.0% (124)	7.4% (862)	2.6% (2,624)
**Level of income**			
(lowest) group 1	27.4% (3,465)	28.4% (3,304)	16.1% (16,589)
group 2	24.7% (3,121)	23.8% (2,773)	17.6% (18,141)
group 3	18.7% (2,370)	22.2% (2,591)	20.8% (21,464)
group 4	16.4% (2,073)	15.2% (1,766)	21.4% (22,072)
(highest) group 5	12.4% (1,574)	9.9% (1,157)	23.7% (24,510)
Missing	0.4% (54)	0.4% (46)	0.5% (505)
**Health status variables**			
CCI score 0–1	61.4% (7,770)	59.6% (6,943)	59.7% (61,670)
CCI score 2–3	14.0% (1,773)	15.0% (1,740)	13.4% (13,803)
CCI score 4–5	19.4% (2,454)	19.7% (2,295)	20.6% (21,311)
CCI score > 6	5.2% (660)	5.7% (660)	6.3% (6,497)
**Municipal social care use**			
Ordinary housing no home-help	82.4% (10,4394	79.8% (9,286)	80.0% (82,577)
Ordinary housing with home-help	11.3% (1,424)	14.5% (1,69	13.8% (14,260)
Municipal care-home	6.3% (799)	5.7% (658)	6.2% (6,444)

*(%) the proportion,

The multivariate logistic models estimating the effect of individual factors on covid-19 mortality for both periods, see table S2. In period 1, men had higher odds than women (OR 1.52, CI:1.38–1.66). Increasing age was strongly associated with increasing odds of covid-19 mortality, as those 75–80 years had (OR 1.96, CI:1.64–2.33) increased risk compared to those 70–74 years, while those 95 + years had a (OR 18.76, CI:15.22–23.13). Those with primary (OR 1.59, CI:1.40–1.79) and secondary (OR 1.41, CI:1.25–1.58) level education had increased odds compared to those with tertiary level education. Individuals born outside of Sweden had higher odds of covid-19 mortality (OR 1.42, CI:1.28–1.58) compared to those born in Sweden. Increased morbidity indicated by CCI score was associated with increased odds of covid-19 mortality (OR 1.24, CI:1.21–1.26). The individual risk factors associated with covid-19 mortality were similar in period 2 with slight differences in effect sizes.

**Fig. 1:**
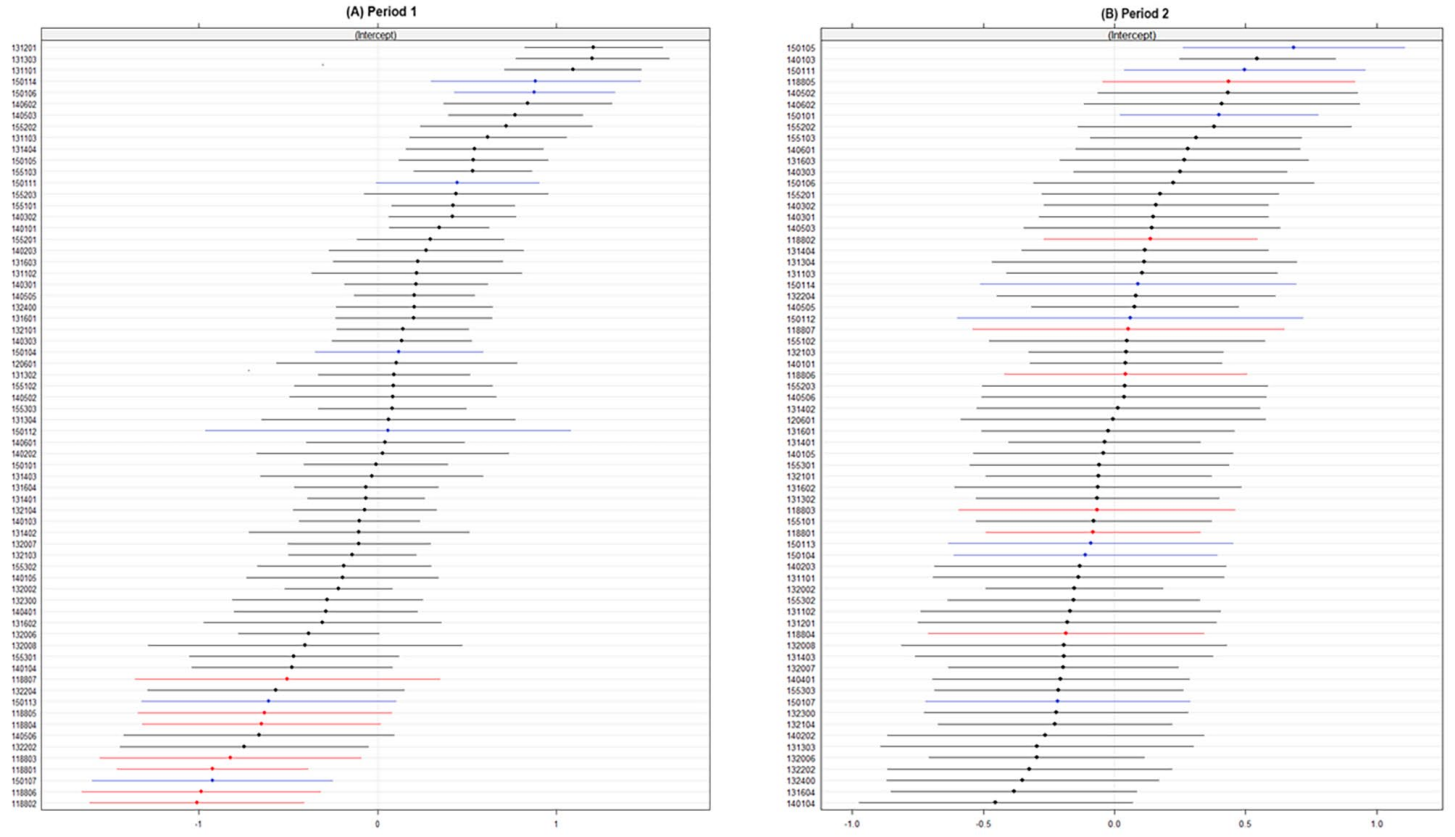
Caterpillar plots of the catchment area specific effects (residuals and CIs) on the risk of covid-19 mortality across the distribution of catchment areas based on the random intercept term from model 0, in period 1 (**A**) and period 2 (**B**), among inhabitants 70 years and older within the municipalities of Norrtälje (the red dots), Södertälje (blue dots) and Stockholm municipality (back dots)

**Fig. 2:**
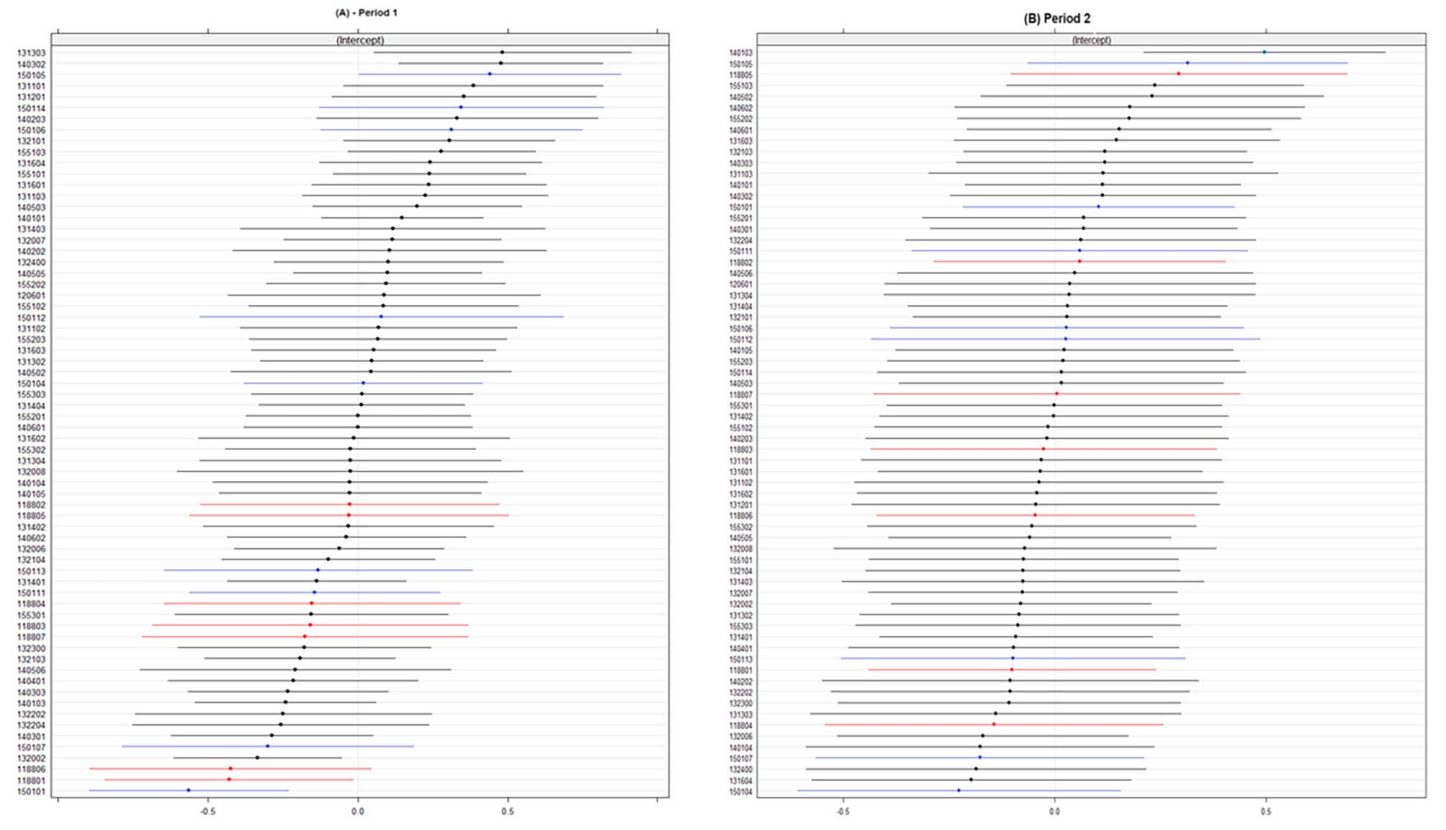
Caterpillar plot of the catchment area specific effects (residuals and CIs) on the risk of covid-19 mortality after adjusting for individual and area-level factors shown in model 3, Tables 3 and 4 for period 1 (**A**) and period 2 (**B**), among inhabitants 70 years and older within the municipalities of Norrtälje (the red dots), Södertälje (blue dots) and Stockholm municipality (black dots)

Figure 1 shows area specific effects of covid-19 mortality across the distribution of catchment areas, the variance was 0.3518 in period 1 and 0.1195 in period 2. In period 1, catchment areas within Norrtälje the red dots were lower across the distribution of covid-19 mortality compared to Södertälje and Stockholm, see Fig. 1(A), however, they were more spread across the distribution in period 2, see Fig. 1(B). Table 3 presents the estimates from the multi-level logistic regression models in period 1. the variance in the probability of covid-19 mortality between catchment areas reduced by 39.3% when controlling for individual factors. Model 2 included both individual and area-level factors, high deprivation (OR:2.09, CI: 1.48–2.96) indicated by NDS and population density (OR 1.21, CI 1.13–1.29) were associated with an increased risk of covid-19 mortality, which explained 67.9% of the variance between catchment areas compared to model 0, see Fig. 2 (A). Model 3 expanded on model 2 and included the area level variable organization of care. There was an increased risk of covid-19 mortality in municipalities with usual care (OR:6.01 CI: 3.26–11.08) in Stockholm, and (OR: 3.37, CI:2.10–6.66) Södertälje compared to IC in Norrtälje, this explained 85.9% of the variance in the risk of covid-19 mortality between catchment areas. The effect of municipal social care was considered in model 4, those living in ordinary housing with home-help (OR:5.93, CI4.88-7.21) and care home residents (OR:22.62, CI:18.73–27.33) had increased risk of covid-19 mortality compared to those without home-help in ordinary housing.

**Table 3:** Multi-level logistic regression analysis of covid-19 mortality among persons 70 years and older in period 1 in the 68 catchment areas within the municipalities of Norrtälje, Södertälje and Stockholm Municipality and variance components of the multi-level regression models

PERIOD 1	Model 1		Model 2		Model 3		Model 4	
	OR	CI 95%	OR	CI 95%	OR	CI 95%	OR	CI 95%
***Individual-level variables***								
Sex (Male)	1.65	(1.45–1.88)	1.66	(1.46–1.89)	1.64	(1.45–1.88)	2.14	(1.87–2.44)
75–80 years	2.04	(1.58–2.61)	2.03	(1.58–2.62)	2.04	(1.58–2.63)	1.63	(1.26–2.10)
80–85 years	4.65	(3.66–5.89)	4.65	(3.67–5.90)	4.65	(3.67–5.91)	2.51	(1.96–3.21)
85–90 years	8.02	(6.32–10.1)	7.99	(6.30-10.14)	8.02	(6.32–10.18)	2.82	(2.19–3.62)
90–95 years	11.96	(9.32–15.3)	11.84	(9.23–15.19)	11.95	(9.31–15.34)	2.85	(2.18–3.72)
95 + years	16.74	(12.4–22.5)	16.45	(12.24–22.12)	16.79	(12.48–22.60)	2.96	(2.16–4.06)
Primary education	1.52	(1.27–1.81)	1.58	(1.33–1.89)	1.53	(1.28–1.83)	1.31	I1.09-1.56)
Secondary education	1.35	(1.14–1.58)	1.37	(1.16–1.62)	1.35	(1.14–1.59)	1.21	(1.02–1.43)
Born outside of Sweden	1.38	(1.18–1.60)	1.39	(1.19–1.61)	1.33	(1.14–1.55)	1.38	(1.19–1.62)
CCI score	1.22	(1.18–1.26)	1.22	(1.19–1.26)	1.22	(1.19–1.26)	1.11	(1.07–1.15)
***Area-level variables***								
**Neighbourhood deprivation score**								
High Deprivation			2.09	(1.48–2.96)	1.98	(1.46–2.67)	1.87	(1.43–2.47)
Moderate Deprivation			1.24	(0.94–1.63)	1.10	(0.89–1.37)	1.02	(0.84–1.23)
Low Deprivation			ref		ref		ref	
**Population Density**			1.21	(1.13–1.29)	1.01	(0.93–1.09)	1.01	(0.94–1.23)
**Organisation of care**								
(Integrated Care) Norrtälje					ref		ref	
(Usual care) Stockholm					6.01	(3.26–11.08)	6.45	(3.68–11.34)
(Usual care) Södertälje					3.37	(2.10–6.66)	3.89	(2.29–6.63)
***Municipal social care use***								
Ordninary housing no home-help							ref	
Ordinary housing with home-help							5.93	(4.88–7.21)
Care home resident							22.62	(18.73–27.33)
	**AIC**	**Variance**	**PCV**	**VPC**				
**Model 0**	12102.3	0.3518	Ref	0.0038325				
**Model 1**	10301.1	0.2134	39.3	0.0057628				
**Model 2**	10279.2	0.1129	67.9	0.0052603				
**Model 3**	10254.9	0.0496	85.9	0.0012675				
**Model 4**	9115.7	0.0217	93.8					

*OR odds ratio, CI confidence interval, Ref = reference, PCV proportional change of the variance, VPC variance partition coefficient, AIC Akaike Information Criterion. *Period 1 included 68 catchment areas and *N* = 127,520 *Model 0: catchment area-specific random intercept of the variation in the probability of covid-19 mortality *Model 1: model 0 + sex (ref = female) + age group (ref 70–74 years) + education level (ref = tertiary education) + country of birth (ref = Sweden) + CCI score. *Model 2: model 1 + neighbourhood deprivation score groups (ref = low NDS least deprived areas) + population density *Model 3: model 2 + Organisation of care (ref=) Integrated Care) Norrtälje * **Model 4**: model 3 + municipal social care use (ref = ordinary housing no home-help). *VPC estimated on the simulation method, model 0 = intercept. Profile 1 = intercept, male, 85–89 years, primary level education, born outside of Sweden, weighted CCI score. Profile 2 = profile 1 + high deprivation area + population density. Profile 3 = profile 2 + municipality with usual care organisation

The VPC reported in Table 3, is based on the following profile (male, 85–89 years, primary education, born outside of Sweden, and an average CCI score) 0.57% of the residual variation is attributable to difference between catchment areas. The VPC decreased slightly when area-level explanatory variables were included 0.52%. Finally, being in a municipality with a usual care the VPC decreased to 0.12% of the residual variation is attributable to differences between catchment areas in period 1.

In period 2, adjusting for individual covariates see Table 4 explained 45.7% of the variation and the addition of area-level factors explained 50.2% of the variation in the distribution of covid-19 mortality across catchment areas. NDS and population density were not significantly associated with risk covid-19 mortality among individuals, and moreover, being in a municipality with usual care was associated with covid-19 mortality in period 2. The addition of area-level variables had a limited effect on the variation of covid-19 mortality between catchment areas in period 2 see Fig. 2(B), and moreover, the VPC estimates show that there was very little residual variation in covid-19 mortality attributable to differences between catchment areas.

**Table 4:** Multi-level logistic regression analysis of covid-19 mortality among person 70 years and older in period 2 in the 68 catchment areas within the municipalities of Norrtälje, Södertälje and Stockholm Municipality and variance components of the multi-level regression models

PERIOD 2	Model 1		Model 2		Model 3		Model 4	
	OR	CI 95%	OR	CI 95%	OR	CI 95%	OR	CI 95%
***Individual-level variables***								
Sex (Male)	1.81	(1.52–2.14)	1.78	(1.51–2.11)	1.78	(1.51–2.11)	2.24	(1.88–2.66)
75–80 years	1.95	(1.39–2.73)	1.95	(1.39–2.73)	1.95	(1.39–2.72)	1.66	(1.18–2.33)
80–85 years	4.61	(3.35–6.33)	4.59	(3.34–6.31)	4.59	(3.34–6.31)	2.87	(2.07–3.98)
85–90 years	8.94	(6.53–12.25)	8.95	(6.54–12.23)	8.03	(6.54–12.23)	4.01	(2.88–5.57)
90–95 years	15.32	(11.07–21.18)	15.42	(11.17–21.31)	15.45	(11.18–21.34)	4.92	(3.48–6.95)
95 + years	26.59	(18.34–38.54)	27.06	(18.68–39.04)	27.06	(18.67–39.20)	6.81	(4.58–10.11)
Primary education	1.52	(1.21–1.90)	1.44	(1.14–1.81)	1.43	(1.14–1.80)	1.28	(1.02–1.61)
Secondary education	1.27	(1.03–1.58)	1.24	(1.00-1.54)	1.24	(0.99–1.53)	1.14	(0.91–1.41)
Born outside of Sweden	1.37	(1.13–1.67)	1.35	(1.00-1.53)	1.36	(1.11–1.65)	1.39	(1.14–1.70)
CCI score	1.23	(1.19–1.28)	1.24	(1.19–1.28)	1.24	(1.19–1.29)	1.15	(1.11–1.21)
***Area-level variables***								
**Neighbourhood Deprivation Score**								
High Deprivation			1.36	(0.97–1.92)	0.95	(0.51–1.77)	1.17	(0.81–1.71)
Moderate Deprivation			1.21	(0.93–1.57)	1.17	(0.67–2.04)	1.12	(0.86–1.45)
Low Deprivation			ref		ref		ref	
**Population Density**			1.00	(0.93–1.05)	0.99	(0.91–1.09)	0.99	(0.91–1.08)
**Organisation of care**								
Integrated Care-Norrtälje					ref		ref	
Usual care-Stockholm municipality					1.25	(0.86–1.83)	1.03	(0.56–1.90)
Usual care-Södertälje					1.2	(0.92–1.56)	1.26	(0.73–2.16)
***Municipal social care use***								
Ordninary housing no home-help							ref	
Ordinary housing with home-help							4.12	(3.25–5.21)
Care home resident							11.9	(9.41–15.05)
	**AIC**	**Variance**	**PCV**	**VPC**				
**Model 0**	7719.1	0.1196	Ref	0.000611927				
**Model 1**	6588.4	0.06492	45.7	0.001077308				
**Model 2**	6588.5	0.0595	50.2	0.001111665				
**Model 3**	6591.5	0.05718	52.2	0.000391568				
**Model 4**	6160.3	0.0468	60.8					

*OR odds ratio, CI confidence interval, Ref = reference, PCV proportional change of the variance, VPC variance partition coefficient, AIC Akaike Information Criterion. *Period 1 included 68 catchment areas and *N* = 123,622 *Model 0: catchment area-specific random intercept of the variation in the probability of covid-19 mortality *Model 1: model 0 + sex (ref = female) + age group (ref 70–74 years) + education level (ref = tertiary education) + country of birth (ref = Sweden) + CCI score. *Model 2: model 1 + neighbourhood deprivation score groups (ref = low NDS least deprived areas) + population density *Model 3: model 2 + Organisation of care (ref=) Integrated Care) Norrtälje * **Model 4**: model 3 + municipal social care use (ref = ordinary housing no home-help). *VPC estimated on the simulation method, model 0 = intercept. Profile 1 = intercept, male, 85–89 years, primary level education, born outside of Sweden, weighted CCI score. Profile 2 = profile 1 + high deprivation area + population density. Profile 3 = profile 2 + municipality with usual care organisation

## Dicussion

The rate of covid-19 mortality among those 70 years and older was lower in Norrtälje compared to the municipalities of Stockholm and Södertälje in period 1, although covid-19 mortality increased in Norrtälje in period 2. Individual risk factors for covid-19 mortality in both periods were male sex, older age, primary education, being born outside of Sweden, and poorer health as indicated by CCI score. Area level risk factors were more important in period 1, as persons in catchment areas with high deprivation, high population density and in municipalities with usual care had an increased risk of covid-19 mortality. Although catchment areas within Norrtälje had lower probability of covid-19 mortality compared to usual care in period 1, the general contextual effects indicate that the relevance of area differences was relatively weak. In period 2, individual risk factors of covid-19 mortality explained more of the variation between catchment areas and the general contextual effects were low, therefore, the area was not as relevant in examining individual differences in covid-19 mortality in period 2.

All persons 70 years and older, irrespective of health status, were identified as a vulnerable group to adverse health outcomes due to covid-19, and recommendations were put in place to protect older people during the covid-19 pandemic [18]. Consistent with previous studies, we observed a strong association between older age and covid-19 mortality [10, 27]. The vulnerability of older people to covid-19 is contributed to by a higher prevalence of multi-morbidity and functional decline [27]. Further, a previous study has shown a positive correlation between CCI score and covid-19 mortality, in which each point increase in CCI score increased the risk of death by 2.5% [28]. The provision of social care is based on needs assessment and those who move into care homes tend to be more frail, have multiple health problems and generally, at greater risk of mortality [29]. Excess mortality due to covid-19 was observed among care home residents compared to individuals living in their own homes during the peak of the covid-19 pandemic [30]. Interventions were introduced to reach vulnerable groups as the Swedish government introduced national restrictions banning visits to care homes on the 1st of April 2020 [18], though some municipalities introduced their own visitation restrictions already in March [13].

The Swedish authorities handling of the covid-19 pandemic was criticised, with concerns about shortages of staff, PPE and testing kits, particularly in municipal social care [13]. In Norrtälje, Tiohundra AB is responsible for the provision and organization of care homes in Norrtälje, which enables more coordination between health-and-social care professionals regarding information sharing which facilitates communication between care professionals [15, 16]. Additionally, the shared funding in Norrtälje supported an effective supply chain for PPE, testing kits and other resources [13]. This aspect of IC could have contributed to the lower covid-19 mortality among municipal social care users in Norrtälje compared to other municipalities in period 1. However, a study found that the proportion of covid-19 infections among care home residents was mostly the same or slightly higher compared to infectious among the care staff in Nordic countries [4]. A systematic review examining the characteristics of care home facilities and staff on risk of infectious disease transmission highlighted that larger facilities in urban areas, with temporary staff or staff residing in highly infectious areas, had an increased risk of transmission in facilities [31]. This might elucidate why covid-19 mortality was higher in the usual care settings indicated by the municipalities of Stockholm and Södertälje as these are more urban and densely populated areas compared to Norrtälje.

Congruent with previous studies, we observed that high population density and neighbourhood deprivation were associated with increased risk of covid-19 mortality [2, 10, 30]. High population density is an indicator of community transmission, as covid-19 was primarily spread through face-to-face interactions, unavoidable in densely populated areas, with more people of working age and including essential workers who were still commuting [32]. As Norrtälje has a lower population density compared to Södertälje and Stockholm municipalities, this must be taken into consideration when interpreting the lower covid-19 mortality among inhabitants 70 years in Norrtälje during period 1. Additionally, older persons born outside of Sweden had an increased risk of covid-19 mortality, which is supported by previous studies [6, 8, 10]. There is a concentration of persons born outside of Sweden in socio-economically disadvantaged neighbourhoods compared to neighbourhoods with a higher concentration of Swedish born [33]. Comparatively, Norrtälje has less inhabitants born outside of Sweden, although with a lower SEP compared to Stockholm municipality. Previous studies have observed that individuals with lower SEP had a higher risk of covid-19 mortality [6, 8]. Further, in other European countries a positive association was found between area-level socio-economic disadvantage and covid-19 mortality [34].

### Strengths and limitations

The use of population-based registers to measure individual and area-level risk factors for covid-19 mortality is a strength of this study. Defining area-level based on catchment areas assures an adequate sample size for a multi-level analysis. Distinguishing between the time periods allows for consideration for the differences between the waves of the covid-19 pandemic and the preparedness of the health-and-social care systems to respond [11, 35].

The NBHW reports all cases where the underlying cause of death was covid-19, whether the diagnosis was confirmed via laboratory testing or not [36]. However, discrepancies have been observed between official covid-19 death statistics and the reported underlying and contributing cause-of-death based on a clinical audit in a previous Swedish study [37]. Moreover, this becomes increasingly complicated when ascertaining the underlying cause of death of persons with multiple chronic conditions and limited insight into the process of dying [38]. Using the CCI score to measure morbidity is a strength as the CCI score assesses the number and severity of chronic conditions [22]. Dichotomising country of birth into “Sweden” or “born outside of Sweden” is limitation as it does not consider the diversity of backgrounds or time spent in Sweden.

Additional limitations relate to distinguishing the effect of IC compared to “usual care”. IC systems are often implemented to improve the experience of care for individuals and health-and-social care professionals experience of providing care, along with being compatible with specific needs of the community [39]. The outcome measure of mortality might be considered a crude outcome to assess the experience of care. Moreover, it is difficult to measure whether the provision of care continued as normal during the covid-19 pandemic and what measures were introduced in individual municipalities. The municipalities of Stockholm and Södertälje were considered to have “usual care”. However, to measure what defines usual care is difficult under the unusual circumstances created by the covid-19 pandemic, as ultimately all health-and-social care workers were taking a personal risk to their own health to continue providing good quality care safely while working under extreme pressure [11]. Therefore, it is difficult to ascertain the exact effect of IC, as each municipality has their own funding sources and were the responsible authority who acted as they deemed appropriate during the covid-19 pandemic, though all regions and municipalities must abide by the recommendations introduced nationally [11, 13, 35, 40].

IC might have allowed for better coordination between care providers at the beginning of the covid-19 pandemic systems encountered numerous organisational and logistical challenges. The risk of covid-19 mortality among inhabitants 70 years and older was lower in Norrtälje compared to municipalities with usual care after adjusting for individual and area level risk factors in period (1) However, individual risk factors were more important in period (2) Alternative approaches to organizing care should be considered by policymakers in the future, given that IC approach might help overcome challenges encountered in emergency situations, to better protect and meet the care needs of older people.

### Electronic supplementary material

Below is the link to the electronic supplementary material.

Supplementary Material 1

## Data Availability

The data used to perform this study cannot be made available upon request. In accordance with the General Data Protection Regulation, The Swedish law SFS 2018:218, The Swedish Data Protection Act, the Swedish Ethical Review Act, and the Public Access to Information and Secrecy Act, these types of sensitive data can only be made available after legal review, for researchers who meet the criteria for access to this type of sensitive and confidential data. Readers may contact the first author regarding any further details.

## Abbreviations

Covid-19: Coronavirus Disease
SEP: Socio–economic Position
PPE: Personal protective equipment
IC: Integrated care
RTB: Total population register
LISA: Longitudinal Integrated Database for Social Insurance and Labour Market Studies
NBHW: National Board of Health and Welfare
NPR: National Patient Register
CCI: Charlson Comorbidity Index
OR: Odds Ratio
CI: Confidence Interval
AIC: Akaike Information Criterion
VPC: Variance Partition Coefficient
PCV: Proportional Change in Variance
