# Supplementary material for: Differences in Covid-19 mortality among persons 70 years and older in an integrated care setting in region Stockholm: a multi-level analysis between March 2020-February 2021
Source: BMC Public Health. 2024 Feb 14;24:462. doi: 10.1186/s12889-024-17904-4 (PMC10865543; doi:10.1186/s12889-024-17904-4)
Supplement: Supplementary file 1 — Supplementary Material 1 [file 12889_2024_17904_MOESM1_ESM.docx]

**SUPPLEMENTARY MATERIAL**

Table S1: List of catchment areas

|  | **Catchment Area** | |  |
| --- | --- | --- | --- |
| **Municipality** | **Code** | **Name** | **Inhabitants 70+ years** |
| **Norrtalje** | 118801 | "Norrtälje Norra" | 3448 |
|  | 118802 | "Norrtälje Södra" | 2791 |
|  | 118803 | "Bergshamra/Blidö" | 1277 |
|  | 118804 | "Hallstavik" | 1448 |
|  | 118805 | "Väddö" | 1164 |
|  | 118806 | "Rimbo" | 2007 |
|  | 118807 | "Norrtälje Västra" | 522 |
| **Stockholm** | 120601 | Tureberg | 665 |
|  | 131101 | "Akalla" | 1053 |
|  | 131102 | "Husby" | 874 |
|  | 131103 | "Kista" | 1212 |
|  | 131201 | "Rinkeby" | 928 |
|  | 131302 | "Spånga" | 2217 |
|  | 131303 | "Tensta" | 736 |
|  | 131304 | "Järva" | 589 |
|  | 131401 | "Hässelby Gård" | 4585 |
|  | 131402 | "Hässelby Gård" | 1047 |
|  | 131403 | "Norra Vällingby" | 949 |
|  | 131404 | "Råcksta" | 1773 |
|  | 131601 | "Brommaplan" | 1847 |
|  | 131602 | "Riksby" | 1034 |
|  | 131603 | "Blackeberg-södra Ängby" | 1489 |
|  | 131604 | 6y | 2921 |
|  | 132002 | "Stureplan" | 6372 |
|  | 132006 | "Narvavägen" | 4198 |
|  | 132007 | "Gärdet" | 3058 |
|  | 132008 | "Hjorthagen" | 418 |
|  | 132101 | "Johannes" | 2884 |
|  | 132103 | "Surbrunnsgatan" | 4082 |
|  | 132104 | "Tegnergatan" | 2953 |
|  | 132202 | "S:t Erik" | 1393 |
|  | 132204 | "Västra Kungsholmen" | 1052 |
|  | 132300 | "Matteus" | 1849 |
|  | 132400 | "Odenplan" | 1841 |
|  | 140101 | "Hornstull" | 4322 |
|  | 140103 | "Rosenlund" | 4485 |
|  | 140104 | "Maria/Gamla Stan" | 1953 |
|  | 140105 | "Södra Nämndhuset" | 1663 |
|  | 140202 | "Ringen" | 620 |
|  | 140203 | "Åsö" | 1012 |
|  | 140301 | "Dalen" | 2237 |
|  | 140302 | "Årsta" | 2378 |
|  | 140303 | "Stureby" | 2561 |
|  | 140401 | "Bagarmossen" | 2042 |
|  | 140502 | "Hökarängen" | 1041 |
|  | 140503 | "Sköndal" | 1499 |
|  | 140505 | "Farsta" | 3318 |
|  | 140506 | "Gubbängen" | 975 |
|  | 140601 | "Högdalen" | 2095 |
|  | 140602 | "Rågsved" | 825 |
|  | 155101 | "Fruängen" | 2540 |
|  | 155102 | "Liseberg" | 1157 |
|  | 155103 | "Älvsjö" | 2483 |
|  | 155201 | "Skärholmen" | 1946 |
|  | 155202 | Axelsberg | 888 |
|  | 155203 | "Bredäng" | 985 |
|  | 155301 | "Gröndal" | 1717 |
|  | 155302 | "Axelsberg" | 2111 |
|  | 155303 | "Liljeholmen" | 2358 |
| **Sodertalje** | 150101 | "Luna" | 2746 |
|  | 150104 | "Tallhöjden" | 1686 |
|  | 150105 | "Lina Hage" | 1483 |
|  | 150106 | "Geneta" | 898 |
|  | 150107 | "Järna" | 1829 |
|  | 150111 | "Hovsjö" | 1308 |
|  | 150112 | "Ytterjärna" | 102 |
|  | 150113 | "Rosenborg" | 1124 |
|  | 150114 | "Fornhöjden" | 459 |

**Table S2:** Estimates of individual level variables effect on covid-19 mortality among inhabitants 70 years and older in Stockholm County in period 1 and 2.

| **Period 1** | **Model 0** | | **Model 1** |  |
| --- | --- | --- | --- | --- |
|  | **OR** | **CI** | **OR** | **CI** |
| Sex(Male) | 1.66 | (1.52-1.81) | 1.52 | (1.39-1.66) |
| 75-80 yrs | 2.16 | (1.82-2.57) | 1.96 | (1.64-2.33) |
| 80-85 yrs | 4.77 | (4.04-5.62) | 4.07 | (3.43- 4.81) |
| 85-90 yrs | 10.16 | (8.64–11.93) | 8.22 | (6.96-9.70) |
| 90-95 yrs | 15.54 | (13.10-18.43) | 12.25 | (10.27-14.6) |
| 95+yrs | 23.23 | (18.99–28.43) | 18.76 | (15.21-23.1) |
| Primary education |  |  | 1.59 | (1.40-1.80) |
| Secondary education |  |  | 1.41 | (1.25-1.59) |
| Born outside of Sweden |  |  | 1.43 | (1.28-1.58) |
| CCI score |  |  | 1.24 | (1.21-1.26) |
| **AIC** | 22596.0 |  | 21135.6 |  |
| **Psuedo R-squared** |  |  | 0.635 |  |
| Period 2 | **Model 0** | | **Model 1** | |
|  | **OR** | **CI** | **OR** | **CI 95%** |
| Sex (Male) | 1.83 | (1.63- 2.05) | 1.65 | (1.47-1.87) |
| 75-80 yrs | 1.95 | (1.55-2.45) | 1.75 | (1.40- 2.21) |
| 80-85 yrs | 4.96 | (4.05-6.18) | 4.20 | (3.40- 5.21) |
| 85-90 yrs | 9.31 | (7.59-11.59) | 7.33 | (5.91-9.10) |
| 90-95 yrs | 18.35 | (14.78–22.97) | 13.96 | (11.18-17.44) |
| 95+yrs | 27.94 | (21.54-36.23) | 21.79 | (16.67-28.48) |
| Primary education |  |  | 1.62 | (1.39- 1.89) |
| Secondary education |  |  | 1.27 | (1.09-1.48) |
| Born outside of Sweden |  |  | 1.31 | (1.14-1.51) |
| CCI score |  |  | 1.27 | (1.25-1.30) |
| **AIC** | 14293.6 |  | 13480.6 |  |
| **Psuedo R-squared** | 0.635108 |  | 0.5651573 |  |

*OR=Odds Ration, CI=confidence interval, AIC=Akaike Information Criteria

***Model 0:** Sex (ref=female), Age group (ref=70-75 years)

***Model 1:** Model 0, Education (ref=tertiary education), Country of Birth (ref=Sweden), Weighted CCI score


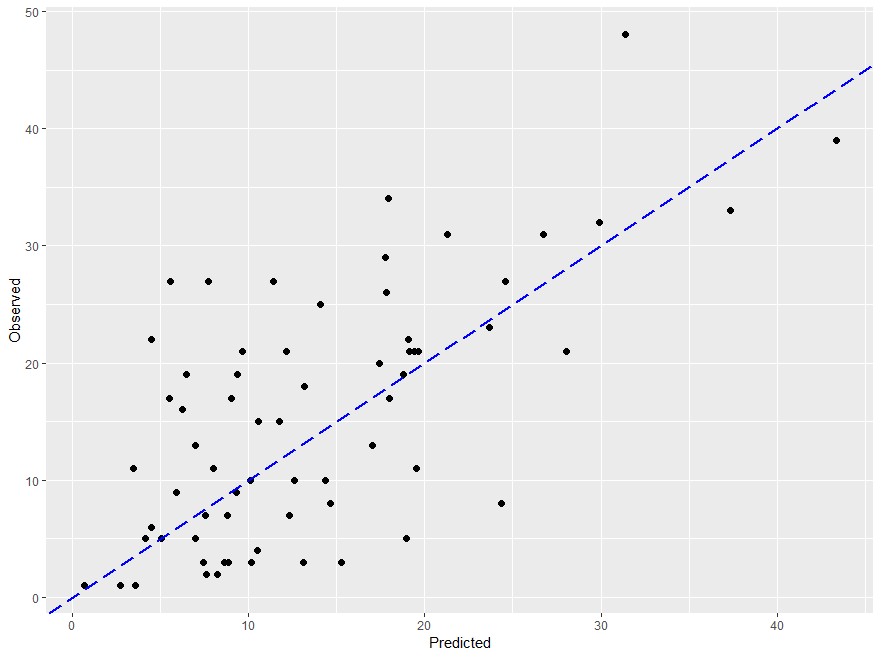


**Figure S1:** Plot of the observed versus predicted values based on the multivariate logistic regression model 1 presented in table S2 for period 1. The corresponding pseudo r-squared=0.635108.


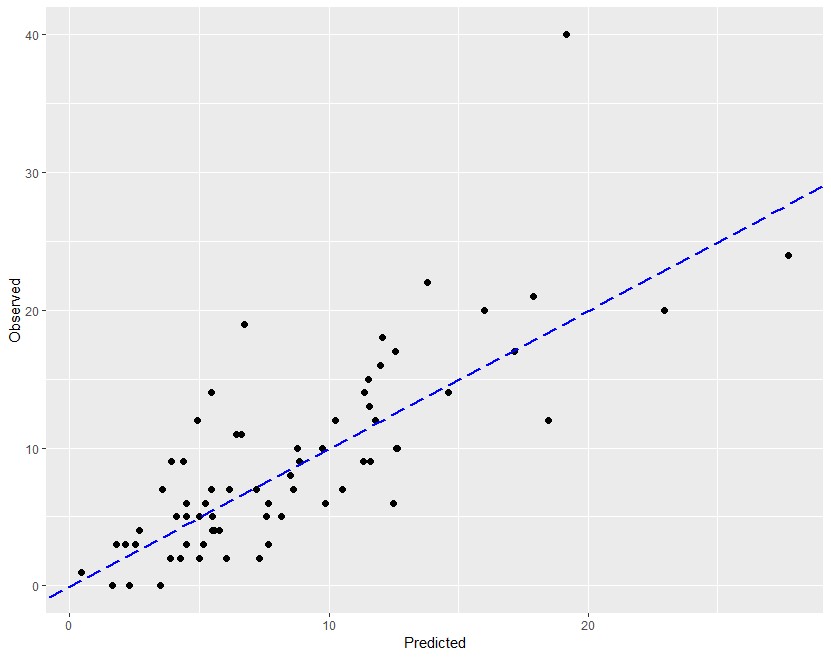


**Figure S2:** Plot of the observed versus predicted values based on the multivariate logistic regression model 1 presented in table S2 for period 2. The corresponding pseudo r-squared= 0.5651573

.

Tables S3: The assessment of the area-level variables in the multi-level logistic regression model

|  | Period 1 | | Period 2 | |
| --- | --- | --- | --- | --- |
|  | **OR** | **CI 95%** | **OR** | **CI 95%** |
| Neighbourhood deprivation score |  |  |  |  |
| High deprivation | 1.42 | (0.98 - 2.05) | 1.29 | 0.97–172 |
| Moderate deprivation | 1.22 | (0.85 - 1.77) | 1.10 | 0.83-1.43 |
| Low deprivation | ref |  | ref |  |
| Population Density | 1.11 | (1.03 - 1.19) | 0.96 | 0.92-1.01 |
| Area organisation of care |  |  |  |  |
| Integrated care (Norrtalje) | ref |  | ref |  |
| Usual care (Stockholm) | 4.57 | (2.68 - 7.78) | 1.21 | 0.93-1.59 |
| Usual care (Sodertalje) | 4.91 | (2.63 - 9.14) | 1.52 | 1.15-2.00 |
|  | **AIC** | **Variance** | **AIC** | **Variance** |
| Model 0 + Municipality organization of care | 12075.8 | 0.1949 | 7715 | 0.0464 |
| Model 0 + Neighbourhood Deprivation Score | 12125.9 | 0.3197 | 7722.3 | 0.1179 |
| Model 0 + Population Density | 12095.1 | 0.2847 | 7723 | 0.1186 |

*OR odds ratio, CI confidence interval, Ref=reference, VPC variance partition coefficient, AIC Akaike Information Criterion. ***Model 0:** catchment area-specific random intercept of the variation in the probability of covid-19 mortality

**Tables S4**: Multi-level models assessing the effect of individual and area level variables added separately on Covid-19 mortality in among persons 70 years and older in period 1.

|  | Model 2 | | Model 3 | | Model 4 | | Model 5 | |
| --- | --- | --- | --- | --- | --- | --- | --- | --- |
|  | **OR** | **(CI 95%)** | **OR** | **(CI 95%)** | **OR** | **(CI 95%)** | **OR** | **(CI 95%)** |
| Individual effects |  |  |  |  |  |  |  |  |
| Sex(Male) | 1.65 | (1.45 - 1.87) | 1.65 | (1.46 - 1.89) | 1.66 | (1.46 - 1.89) | 2.13 | (1.87-2.43) |
| 75-80 yrs | 2.04 | (1.58 - 2.62) | 2.04 | (1.58 - 2.62) | 2.03 | (1.58 - 2.62) | 1.61 | (1.25-2.08) |
| 80-85 yrs | 4.65 | (3.67 - 5.89) | 4.66 | (3.67 - 5.91) | 4.65 | (3.67 - 5.90) | 2.47 | (1.93-3.16) |
| 85-90 yrs | 8.03 | (6.34 - 10.18) | 8.01 | (6.32 - 10.16) | 7.99 | (6.30 - 10.14) | 2.8 | (2.18-3.60) |
| 90-95 yrs | 12.01 | (9.36 - 15.38) | 11.89 | (9.26 - 15.25) | 11.84 | (9.23 - 15.19) | 2.84 | (2.18-3.71) |
| 95+yrs | 16.81 | (12.51 - 22.59) | 16.58 | (12.33 - 22.29) | 16.45 | (12.24 - 22.12) | 2.98 | (2.18-4.07) |
| Primary education | 1.51 | (1.26 - 1.81) | 1.56 | (1.31 - 1.87) | 1.58 | (1.33 - 1.89) | 1.29 | (1.02-1.42) |
| Secondary education | 1.34 | (1.14 - 1.58) | 1.36 | (1.16 - 1.61) | 1.37 | (1.16 - 1.62) | 1.29 | (1.08-1.55) |
| Born outside of Sweden | 1.37 | (1.18 - 1.59) | 1.38 | (1.19 - 1.61) | 1.39 | (1.19 - 1.61) | 1.41 | (1.21-1.65) |
| CCI score | 1.22 | (1.19 0 - 1.26) | 1.22 | (1.19 - 1.26) | 1.22 | (1.19 - 1.26) | 1.11 | (1.08-1.15) |
| *Specific contextual effects* |  |  |  |  |  |  |  |  |
| Neighbourhood deprivation score | |  |  |  |  |  |  |  |
| High deprivation | 1.16 | (0.83 - 1.60) |  |  |  |  |  |  |
| Moderate deprivation | 1.09 | (0.73 - 1.38) |  |  |  |  |  |  |
| Low deprivation | ref |  |  |  |  |  |  |  |
| Population Density |  |  | 1.11 | (1.04 - 1.18) |  |  |  |  |
| Care organisation |  |  |  |  |  |  |  |  |
| Integrated care (Norrtalje) |  |  |  |  | ref |  |  |  |
| Usual care (Stockholm) |  |  |  |  | 4.16 | (2.59 - 6.66) |  |  |
| Usual care (Sodertalje) |  |  |  |  | 4.14 | (2.39 - 7.15) |  |  |
| Municipal social care use |  |  |  |  |  |  |  |  |
| independent no home-help |  |  |  |  |  |  | ref |  |
| Home-help user |  |  |  |  |  |  | 5.96 | 4.91-7.25 |
| Care home residents |  |  |  |  |  |  | 22.52 | 18.64-27.19 |
|  | **AIC** | **Variance** | **AIC** | **Variance** | **AIC** | **Variance** | **AIC** | **Variance** |
| Model 0 | 12102.3 | 0.3518 | 12102.3 | 0.3518 | 12102.3 | 0.3518 | 12102.3 | 0.3518 |
| Adjusted | 10304.1 | 0.2076 | 10291.7 | 0.174 | 10270.2 | 0.09256 | 9373.1 | 0.188 |

***OR** odds ratio, **CI** confidence interval, **Ref** reference, V**ariance** the individual variance in the probability of covid-19 mortality distributed across the catchment areas. **PCV** proportional change of the variance, AIC Akaike Information Criterion. *Period 1 included 68 catchment areas and N=127,520 ***Model 0:** catchment area-specific random intercept of the variation in the probability of covid-19 mortality ***Model 1**=model 0 + individual factors (sex, age group, level of education, country of birth, weighted and CCI score. ***Model 2**=Model 1 + Neighbourhood deprivation score (ref=least deprived). ***Model 3**= Model 1 + population density. \***Model 4**=Model 1 + organisation of care in municipality (ref=integrated care in Norrtälje. ***Model 5=** Model 1 + municipal social care use (ref=ordinary housing without hom-help.

**Table S5:** Multi-level models assessing the effect of individual and area level variables added separately on Covid-19 mortality among persons 70 years and older in period 2.

|  | **Model 1** | | **Model 2** | | **Model** 3 | | **Model** 4 | | **Model** 5 | |
| --- | --- | --- | --- | --- | --- | --- | --- | --- | --- | --- |
|  | **OR** | **(CI 95%)** | **OR** | **(CI 95%)** | **OR** | **(CI 95%)** | **OR** | **CI 95%** | **OR** | **CI 95%** |
| ***Individual factors*** |  |  |  |  |  |  |  |  |  |  |
| Sex (male) | 1.81 | 1.52 - 2.14 | 1.79 | 1.51 - 2.12 | 1.78 | 1.51 - 2.11 | 1.79 | 1.51 - 2.12 | 2.26 | 1.90-2.68 |
| 75-80 years | 1.95 | 1.39 - 2.73 | 1.94 | 1.37 - 2.72 | 1.95 | 1.30 - 2.73 | 1.94 | 1.39 - 2.73 | 1.66 | 1.18-2.33 |
| 80-85 years | 4.61 | 3.35 - 6.33 | 4.59 | 3.34 - 6.31 | 4.60 | 3.35 - 6.32 | 4.60 | 3.34 - 6.32 | 2.88 | 2.08-3.99 |
| 85-90 years | 8.94 | 6.53 - 12.25 | 8.95 | 6.55 - 12.23 | 8.94 | 6.54 - 12.23 | 8.95 | 6.54 -12.24 | 4.01 | 2.87-5.56 |
| 90-95 years | 15.32 | 11.07-21.18 | 15.46 | 11.19 -21.35 | 15.42 | 11.16 - 21.31 | 15.40 | 11.14-21.28 | 4.88 | 3.46-6.90 |
| [5+ years | 26.59 | 18.34 - 38.54 | 26.92 | 18.59-39.0 | 27.05 | 18.67 - 39.19 | 26.84 | 18.53-38.89 | 6.68 | 4.50-9.92 |
| Primary education | 1.52 | 1.21 - 1.90 | 1.47 | 1.17 - 1.85 | 1.43 | 1.14 - 1.81 | 1.48 | 1.18 - 1.86 | 1.34 | 1.07-1.68 |
| Secondary education | 1.27 | 1.03 - 1.58 | 1.26 | 1.02 - 1.56 | 1.24 | 0.99 - 1.53 | 1.26 | 1.02 - 1.68 | 1.16 | 0.94-1.44 |
| Born outside of Sweden | 1.37 | 1.13 - 1.67 | 1.38 | 1.13 - 1.67 | 1.35 | 1.09 - 1.65 | 1.39 | 1.13 - 1.68 | 1.41 | 1.16-1.71 |
| CCI score | 1.23 | 1.19 - 1.28 | 1.24 | 1.19 - 1.28 | 1.23 | 1.19 - 1.28 | 1.24 | 1.19 - 1.28 | 1.15 | 1.10-1.20 |
| ***Area Level factors*** |  |  |  |  |  |  |  |  |  |  |
| **Oraganisation of care** |  |  |  |  |  |  |  |  |  |  |
| Integrated care (Norrtalje) |  |  | ref |  |  |  |  |  |  |  |
| Usual care (Stockholm) |  |  | 0.83 | (0.59 - 1.17) |  |  |  |  |  |  |
| Usual care (Sodertalje) |  |  | 1.16 | (0.75 - 1.80) |  |  |  |  |  |  |
| **Area Deprivation score** | | | | | | | | | | |
| Low deprivation |  |  |  |  | ref |  |  |  |  |  |
| High deprivation |  |  |  |  | 1.39 | (1.06 - 1.82) |  |  |  |  |
| Moderate deprivation |  |  |  |  | 1.21 | (0.94 - 1.56) |  |  |  |  |
| **Population Density** |  |  |  |  |  |  | 0.96 | (0.92 - 1.01 |  |  |
| **Municipal Social care use** | | | | | | | | | | |
| Independent no home-help |  |  |  |  |  |  |  |  | ref |  |
| Ordinary house with home-help |  |  |  |  |  |  |  |  | 4.13 | 1.26-5.23 |
| Care home resident |  |  |  |  |  |  |  |  | 11.97 | 9.46-15.13 |
|  | **AIC** | **Variance** | **AIC** | **Variance** | **AIC** | **Variance** | **AIC** | **Variance** | **AIC** | **Variance** |
| Unadjusted model | 7719.1 | 0.1196 | 7719.1 | 0.1196 | 7719.1 | 0.1196 | 7719.1 | 0.1196 | 7719.1 | 0.1196 |
| Adjusted model | 6588.4 | 0.06492 | 6586.5 | 0.05913 | 6588 | 0.05692 | 6587.9 | 0.06451 | 6155.1 | 0.05082 |

***OR** odds ratio, **CI** confidence interval, **Ref** reference, V**ariance** the individual variance in the probability of covid-19 mortality distributed across the catchment areas. **PCV** proportional change of the variance, AIC Akaike Information Criterion. *Period 1 included 68 catchment areas and N=123,622 ***Model 0:** catchment area-specific random intercept of the variation in the probability of covid-19 mortality ***Model 1**=model 0 + individual factors (sex, age group, level of education, country of birth, weighted and CCI score. ***Model 2**=Model 1 + Neighbourhood deprivation score (ref=least deprived). ***Model 3**= Model 1 + population density. \***Model 4**=Model 1 + organisation of care in municipality (ref=integrated care in Norrtälje. ***Model 5=** Model 1 + municipal social care use (ref=ordinary housing without hom-help.

Table S6: Estimates of the variance partition coefficient estimated via simulation method for a range

profiles specified based on values of the individual-and-area level variables.

| **Period 1** | | | | | |
| --- | --- | --- | --- | --- | --- |
|  | **Profile 0** | **Profile 1** | **Profile 2** | **Profile 3** | **Profile 4** |
| **Model 1** | 0.000252 | 0.005763 | 0.0008391 | 0.0046705 | 0.0057628 |
| **Model 2** | 0.000143 | 0.005260 | 0.0003833 | 0.0025913 | 0.0052603 |
| **Model 3** | 0.000107 | 0.000806 | 0.0001815 | 0.0012099 | 0.0012675 |
| **Period 2** | | | | | |
|  | **Profile 0** | **Profile 1** | **Profile 2** | **Profile 3** | **Profile 4** |
| **Model 1** | 0.000037065 | 0.00077308 | 0.0001133161 | 0.0007720136 | 0.001077308 |
| **Model 2** | 0.00003619248 | 0.001111665 | 0.0000891532 | 0.000696059 | 0.001111665 |
| **Model 3** | 0.00004317289 | 0.00149605 | 0.001022411 | 0.001009644 | 0.0009391568 |

The profiles based on the explanatory variables included in **model 1:*Profile 1:** constant, male, 85-89 years, primary education, born in Sweden, CCI score. ***Profile 2:** constant, female, 75-79 years, secondary education, born outside of Sweden, CCI score. ***Profile 3:** constant, male, 80-84 years, primary education, born outside of Sweden, CCI score. ***Profile 4:** constant, male, 85/89 years, secondary education, born in Sweden, CCI score

The profiles based on the explanatory variables included in **model 2: *Profile 0:** constant, other variables 0**. *Profile 1:** constant, male, 85-89 years, primary education, born in Sweden, CCI score, high deprivation, population density. ***Profile 2:** constant, female, 75-79 years, secondary education, born outside of Sweden, CCI score, low deprivation, population density. ***Profile 3**: constant, male, 80-84 years, primary education, born outside of Sweden, CCI score, moderae deprivation, population density. ***Profile 4:** constant, male, 85/89 years, secondary education, born in Sweden, CCI score, high deprivation, population density

The profiles based on the explanatory variables included in **model 3: *Profile 0:** constant, other variables 0 ***Profile 1**: constant, male, 85-89 years, primary education, born in Sweden, CCI score, high deprivation, population density. usual care organization. ***Profile 2:** constant, female, 75-79 years, secondary education, born outside of Sweden, CCI score, low deprivation, population density. usual care organization. ***Profile 3:** constant, male, 80-84 years, primary education, born outside of Sweden, CCI score, moderate deprivation, population density, usual care organization. ***Profile 4:** constant, male, 85/89 years, secondary education, born in Sweden, CCI score, high deprivation, population density, usual care organization.

**,**
